# “Social Media Misinformation”—An Epidemic within the COVID-19 Pandemic

**DOI:** 10.4269/ajtmh.20-0592

**Published:** 2020-06-12

**Authors:** Mohammed Yaseen Ahmed Siddiqui, Kamran Mushtaq, Mouhand F. H. Mohamed, Hussam Al Soub, Mohamed Gaafar Hussein Mohamedali, Zohaib Yousaf

**Affiliations:** Department of Medicine; Hamad Medical Corporation; Doha, Qatar; E-mail: msiddiqui2@hamad.qa; Dresden International University (DIU); Dresden, Germany; Department of Gastroenterology and Hepatology; Hamad Medical Corporation; Doha, Qatar; E-mail: bkamrans@hotmail.com; Department of Medicine; Hamad Medical Corporation; Doha, Qatar; Dresden International University; Dresden, Germany (DIU); E-mail: dr.m.oraibny@hotmail.com; Department of Infectious Disease; Hamad Medical Corporation; Doha, Qatar; Weill Cornell Medicine (WCM-Q); Doha, Qatar; E-mail: halsoub@hamad.qa; Department of Medicine; Hamad Medical Corporation; Doha, Qatar; E-mail: mmomhamedali@hamad.qa; Department of Medicine; Hamad Medical Corporation; Doha, Qatar; Dresden International University; Dresden, Germany (DIU); E-mail: zohaib.yousaf@gmail.com

Dear Editor,

We read with great interest the recently published perspective “Erroneous Communication Messages on COVID-19 in Africa.” In his perspective, Seytre^1^ writes about the importance of miscommunication and how it affects society’s attitudes. He goes on to discuss lingering mistrust generated by misinformation during the Ebola epidemic and its lasting impact on control of the COVID-19 pandemic. Social media has penetrated every sphere of our lives. Facebook, Twitter, Instagram, and blogs impact our thinking patterns, beliefs, and mental health. We concur with the author about the impact of miscommunication on society’s mental, physical, and social fabric. In addition, we would like to highlight the personal toll it can take on individual members of any community. We are sharing two cases to highlight the real-world implications of social media misinformation during the current COVID-19 pandemic.

Two middle-aged South Asian men of low socioeconomic status, living in separate shared housing, were exposed to COVID-19–positive contacts. Both patients presented to a designated COVID-19 treatment facility in Qatar after ingesting chemical substances. They had no past medical or psychiatric illnesses. The first man ingested about 15 mL of a surface disinfectant but did not report any symptoms. The second man experienced multiple episodes of non-bilious vomiting after ingesting approximately 100 mL of alcohol-based hand sanitizer. Apart from mild derangement in their transaminases, other laboratory tests were unremarkable. Both patients tested positive for COVID-19, and both, fortunately, had an unremarkable clinical course. These men ingested the disinfectant and sanitizer based on a firm belief that it would protect them from SARS-COV-2 infection, built on social media advice.

Unvetted information is freely available on social media. Opinion pieces are perceived as facts. There has been a perpetual stream of news on the pandemic, creating a sense of urgency and anxiety. Repeated exposure to this stream of misinformation may affect the construct of external reality. This may lead to a delusion-like experience, which has been linked to anxiety and social media overuse.^2,3^ Social isolation has tipped the balance of relationships and emotional connections from real to virtual for many. Indeed, we are in a virtual, long-term, emotionally charged relationship of sorts with social media. This relationship has led to a delusion-like experience, affecting multiple people separated by space and time, with social media as the common denominator.^4–6^

The two described cases are just the tip of the iceberg of a “hidden epidemic” of nonevidence-based medical advice regarding COVID-19 that is rampant on social media, and not limited by geographic, religious, cultural, or socioeconomic boundaries. This “epidemic” adds to the strain of the pandemic on medical and psychological healthcare resources. It is incumbent on us to fight this social misinformation epidemic, before it turns into another pandemic.
